# Predicting Stability of Barley Straw-Derived Biochars Using Fourier Transform Infrared Spectroscopy

**DOI:** 10.1021/acssusresmgt.4c00148

**Published:** 2024-08-16

**Authors:** Monica A. McCall, Jonathan S. Watson, Mark A. Sephton

**Affiliations:** † Earth Science and Engineering, Imperial College London, Exhibition Rd, South Kensington, London SW7 2BX, United Kingdom; ‡ Grantham Institute for Climate Change and the Environment, Imperial College London, South Kensington, London SW7 2AZ, United Kingdom

**Keywords:** biochar, Fourier-transform infrared spectroscopy, partial least squares regression, stability, barley straw

## Abstract

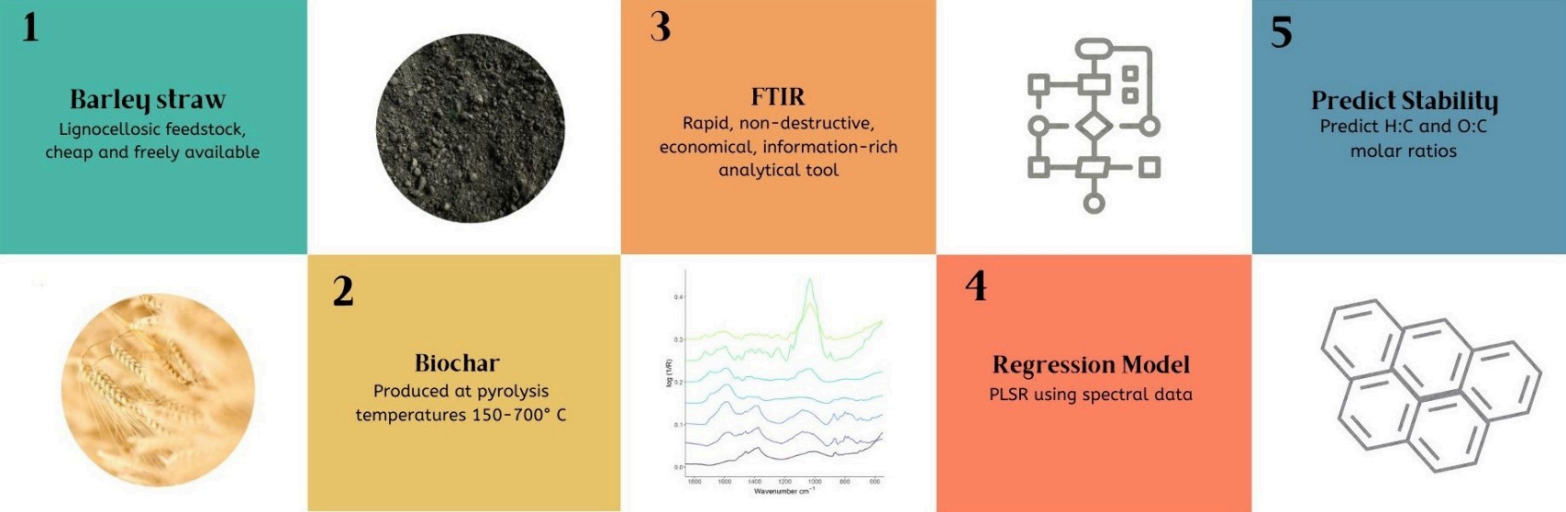

In order to estimate the ability of biochar to sequester carbon as part of greenhouse gas removal technology, there is a need for rapid and accessible estimations of biochar stability. This study employs a novel method using Fourier transform infrared spectroscopy (FTIR) to predict common stability indicators, namely H:C and O:C molar ratios. Biochars derived from barley straw were produced at temperatures from 150 to 700 °C. The greatest compositional changes of the biochars occurred between 200 and 400 °C. All biochars produced at ≥400 °C achieved H:C < 0.7 and O:C < 0.4, indicative of biochars suitable for soil application. Regression models were built using FTIR data to predict H:C and O:C molar ratios. The H:C model produced a coefficient of determination (*R*^2^) of 0.99, mean absolute percentage error (MAPE) 6.86%, and root-mean-square error (RMSE) of 0.07. The O:C model achieved the same *R*^2^ (0.99), MAPE of 9.02%, and RMSE of 0.03. Our results demonstrate that combining FTIR data with modeling is a promising rapid and accessible method for attaining biochar stability data.

## Abstract 2

This research investigates a new method to predict stability data of biochar, a material used in greenhouse gas removal and soil amendment.

## Introduction

Greenhouse gas emissions have indisputably caused widespread changes to our climate and life on Earth.^1^ Biochar, the carbon-rich and environmentally recalcitrant product of biomass pyrolysis, can be a useful part of greenhouse gas removal (GGR) technologies.^2^ Carbon in biochar is estimated to have a mean residence time (MRT) on centennial^3,4^ to millennial time scales.^5,6^ In 2022, biochar production amounted to 0.5 MtCO_2_ yr^–1^, 0.03% of global total carbon dioxide removal (CDR), and by 2030 this is projected to increase to 65 MtCO_2_ yr^–1^.^7^

When used as a soil amendment, biochar has several benefits including increased water retention and nutrient holding capacity,^8^ increased plant growth,^9^ reduction in mineralization of soil organic matter,^10^ and lower greenhouse gas emissions from the soil.^11^ Indirect climate mitigation effects can also be realized by a reduced demand on irrigation^12^ and fertilizer.^13^ Biochar characteristics in soil are overwhelming correlated to pyrolysis temperatures and feedstock types.^14^ Biochar is commonly made from lignocellulosic biomass, which primarily comprises the biopolymers cellulose, hemicellulose, and lignin.^15^ Biochar derived from barley straw, a lignocellulosic feedstock, has been investigated in several studies.^16−19^ Barley straw is chemically well-characterized in the literature,^19^ inexpensive (at £239–£276 per tonne in 2022),^20^ and easily accessible, with about 1.8 million tonnes per year available for pyrolysis in the UK.^21^

The most decisive parameter to estimate the carbon sequestration potential of biochar is its stability.^22^ Currently, the most widely accepted stability indicators are molar ratios of H:C_org_ and O:C_org_, both of which are used as proxies for the degrees of unsaturation and condensation.^23^ Guidelines from the International Biochar Initiative (IBI),^23^ European Biochar Certificate (EBC),^24^ and carbon-trading platforms^25−27^ require H:C_org_ ratios <0.7, and sometimes additionally an O:C_org_ ratio <0.4,^25^ for biochar to be certified and later used in carbon offsetting schemes. Though the use of molar ratios is accurate and conservative to determine biochar stability, their acquisition is expensive, resource intensive, destructive, and requires technical expertise. There is a need for a method that provides a rapid and accessible estimation of the biochar stability.

Fourier transform infrared spectroscopy (FTIR) is commonly used to qualitatively investigate the presence or absence of functional groups in biochar.^28−30^ FTIR is particularly advantageous as it is non-destructive, rapid, information-rich, and economical. Very few studies^31,32^ have employed the use of FTIR quantitatively for biochar characterization. However, there are limited studies that use near-infrared (NIR) spectroscopy, a related technique, to estimate biochar stability by using spectra to build regression models.^33−35^ In this proof-of-concept study, we propose the quantitative use of FTIR as a novel method for predicting biochar stability using a well-characterized feedstock, barley straw. Our method is implemented by using regression models to predict molar H:C and O:C ratios from the infrared spectra of barley straw-derived biochar at various pyrolysis temperatures. Results will allow for more effective monitoring of biochar in production and rapid accessibility to stability information.

## Materials and Methods

### Biochar Production

Barley straw was sourced from Rookery Farm Hinton-in-the-Hedges, Brackley, Northamptonshire, UK. The barley straw was first homogenized into a powder, weighed, and then pyrolyzed in a Carbolite Gero tube furnace inside a Coors high alumina combustion boat. The pyrolysis temperature treatments ranged from 150 to 700 °C in increments of 50 °C, with heating and cooling rates of 5 °C/min and a residence time of 30 min at the peak temperature. Nitrogen gas was used at a flow rate of 1 L/min to purge the tube and combustion boat of any oxygen. The resultant residues, i.e., the biochar, were weighed and homogenized in a mortar and pestle.

### FTIR Specifications

The biochars were analyzed by attenuated total reflectance-FTIR (ATR-FTIR) on a Nicolet 5700 FTIR spectrometer using 128 scans. Six replicates of each biochar sample were acquired at a resolution of 4 cm^–1^ (4000 to 400 cm^–1^). The unpyrolyzed barley straw was also analyzed by FTIR, as well as standards of kraft lignin and cellulose for comparison. The resulting spectra were trimmed to 4000–550 cm^–1^ and then baselined in Spectragryph software using the advanced adaptive baseline, which applies a moving-average smoothing with an interval size of 15.^36^

### Elemental Analysis

Elemental analysis was performed by Iso-Analytical Limited. Briefly, 1.5 ± 0.1 mg of each biochar sample was oven-dried for 3 days at 60 °C to remove moisture. Analysis was undertaken using a Europa Scientific elemental analyzer–isotope ratio mass spectrometer (EA-IRMS). Twenty percent of samples were duplicated, and elemental data of replicates were averaged.

### Modeling

Statistical analysis and modeling were completed using the pls package in R, version 3.4.0. The FTIR spectral files (*n* = 78) were randomized and then split into a training set with 80% of the data, while the other 20% were used as test data. Cross-validation, which can be used to assess the model’s performance on unseen data,^37^ was performed on the training set to determine the optimal number of components to retain in the model without overfitting. The leave-one-out cross-validation method was chosen, which trains an iterative model on a dataset that contains *n* – 1 observations and uses the left-out observation as a test set. It is typically the most appropriate cross-validation method for smaller data sets.^38^

**Figure 1:**
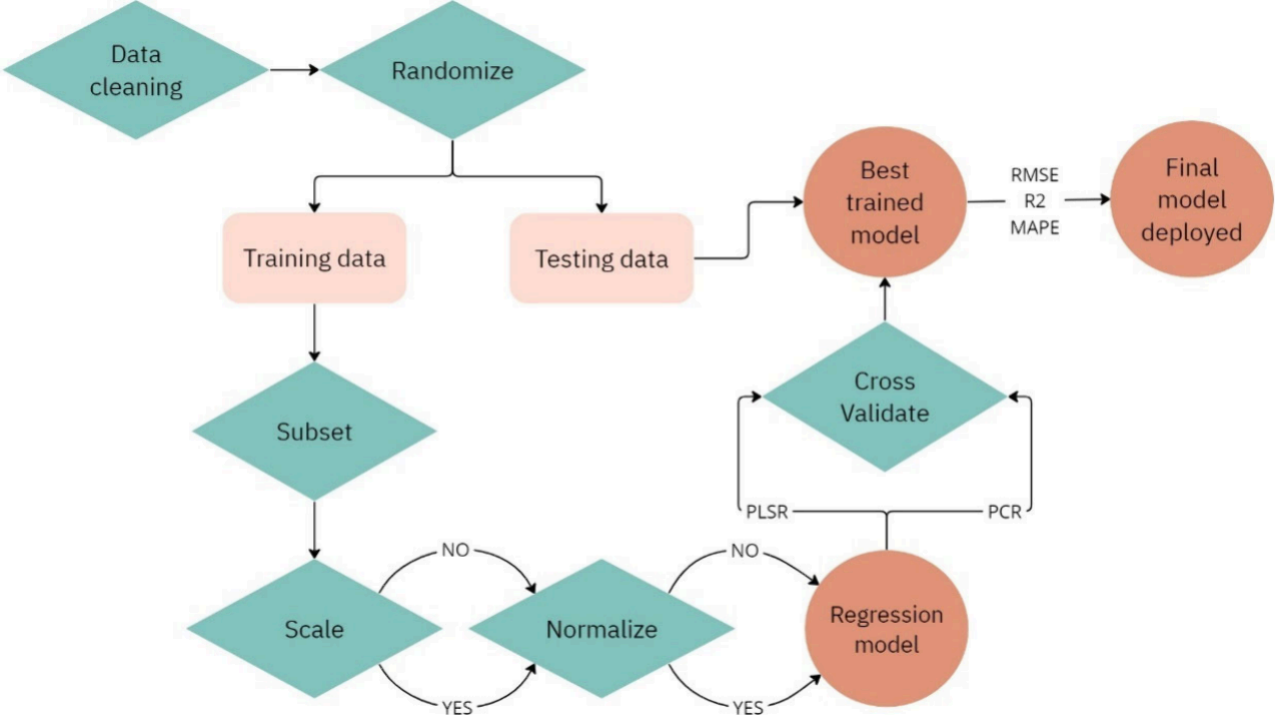
Flow chart of steps used to create the models. Rectangles denote data, diamonds signify actions, and circles are model versions.

Several steps for pre-processing of the model were assessed, such as sub-setting, normalization, and scaling. Three different subsets of the spectra were tested in the model: the whole spectrum, the fingerprint region (1800–700 cm^–1^), and a “cleaned” version where the 2500–1800 cm^–1^ region, usually containing noise from atmospheric carbon dioxide, was removed.^39^ Initial tests included second derivative pre-processing of the spectral data, but this was ruled out owing to a considerable increase in noise. In the pls package, scaling was comprised of standardizing each predictor variable by dividing by its standard deviation.^40^ In order to predict molar ratios, the model tested both partial least squares regression (PLSR) and principal component regression (PCR), both multivariate regression methods particularly useful in circumstances where there are several predictor variables, limited observations, and possible presence of multicollinearity.^40^ The major difference between the two approaches is that PLSR is supervised, whereas PCR is unsupervised. The algorithms for both PLSR and PCR are described in the literature.^40^ The model iterations that minimized root mean square error (RMSE) and mean absolute percent error (MAPE) while maximizing the coefficient of determination (*R*^2^) were considered best performing. Ease of pre-processing and consistency between H:C and O:C model algorithms were also considered. The loadings of model predictions were then investigated. A flow chart of steps used in pre-processing, building, and selecting the models is illustrated in Figure 1.

## Results and Discussion

### Mass Loss and Elemental Data

**Figure 2:**
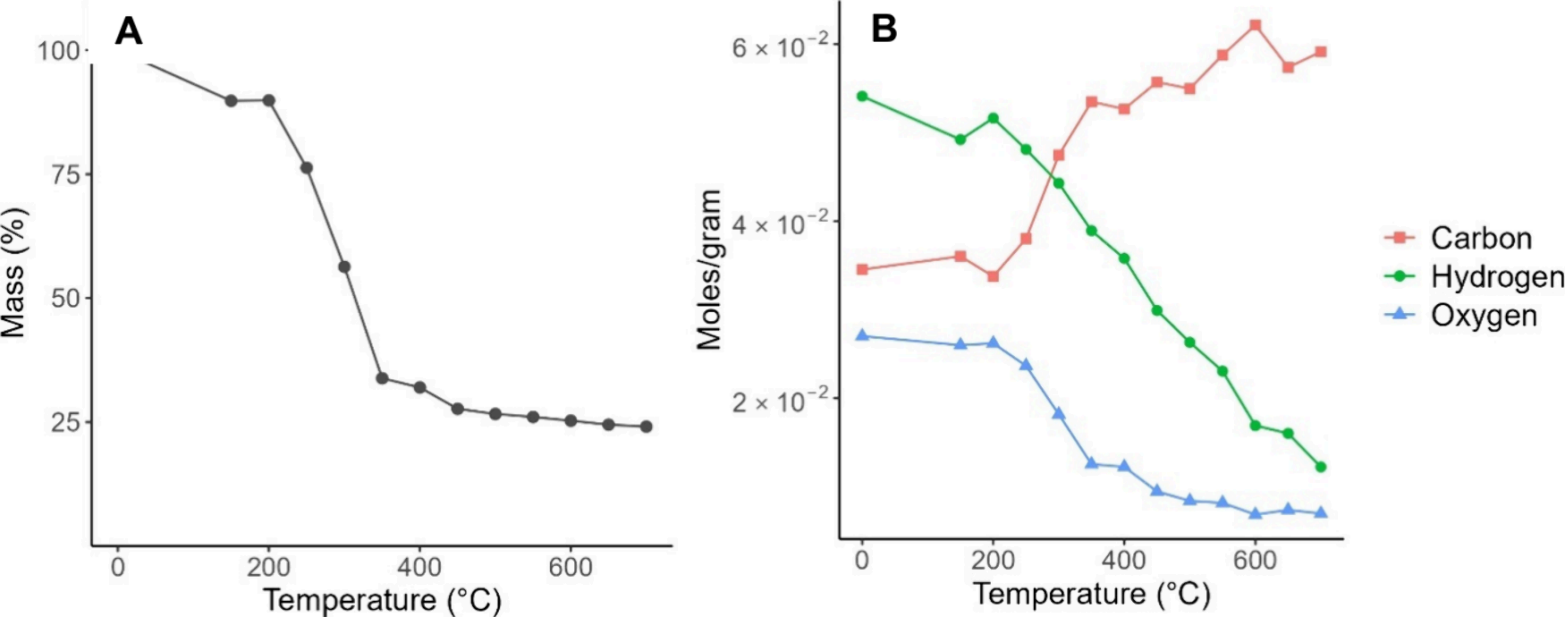
(A) Mass percentage of barley straw-derived biochar relative to the unpyrolyzed starting material, at various pyrolysis temperatures. (B) Carbon, hydrogen, and oxygen elemental content as determined by elemental analysis for each pyrolysis temperature treatment of barley straw, expressed in moles per gram.

The percent mass loss and elemental composition at each respective pyrolysis temperature are summarized in Figure 2.

The greatest rate of mass loss occurs from 200 to 350 °C, a substantial reduction from 90% to 34% of initial mass, as demonstrated in Figure 2A. This is followed by limited loss between 350 and 450 °C, from 34% to 28%, and a plateau thereafter until 700 °C, to a final mass 24% of the unpyrolyzed barley straw. Previous workers^41^ have observed similar results with biochar derived from pine wood, pine needles, and fescue grass.

Figure 2B illustrates a clear trend of increasing carbon corresponding to a decrease in hydrogen and oxygen content with temperature. Beginning at 200 °C, hydrogen content declines at a constant rate from 0.052 mol/g to a minimum of 0.012 mol/g at 700 °C. Initially, oxygen also decreases rapidly for biochars produced at 200 to 350 °C, halving from 0.026 to 0.013 mol/g. However, oxygen then continues a more gradual decline from 400 to 700 °C to a final concentration of 0.007 mol/g. This could be explained by more hydrogen-containing volatiles being removed, such as methane and hydrogen gas.^18^ These trends are clearly mirrored in the carbon content, which increases rapidly from 0.034 mol/g at 200 °C to 0.053 mol/g at 350 °C and steadily increases at higher temperatures. A very similar trend of decreasing O and H in favor of increasing C in wood-derived biochar from 70 °C to 350 °C has been reported in the literature.^42^ Interestingly, the carbon content at 600 °C is the highest of those at all temperatures, though the lowest hydrogen and oxygen contents corresponded with 700 °C. This could indicate the more widespread breaking of C–C bonds beyond 600 °C or could be attributed to error in the elemental analysis.

The trends in elemental composition, particularly the dramatic reductions of oxygen and hydrogen and increase in carbon content from 200 to 350 °C, coincide with the reduction in mass in Figure 2A, implying the mass loss in this temperature range is primarily attributed to loss of oxygen- and hydrogen-containing volatiles, including water, carbon monoxide, carbon dioxide, hydrogen gas, and methane.^18^ Though the biochar masses do not severely change with increasing pyrolysis temperatures above 400 °C, Figure 2B suggests that the composition of the biochars is becoming substantially more aromatized and condensed. Therefore, mass loss alone cannot be considered a reliable predictor of carbon content in biochar. Lastly, owing to the rate of aromatization decreasing after 400 °C, these results suggest that biochar production above this temperature is diminishingly cost-effective.

#### Van Krevelen Diagram

**Figure 3:**
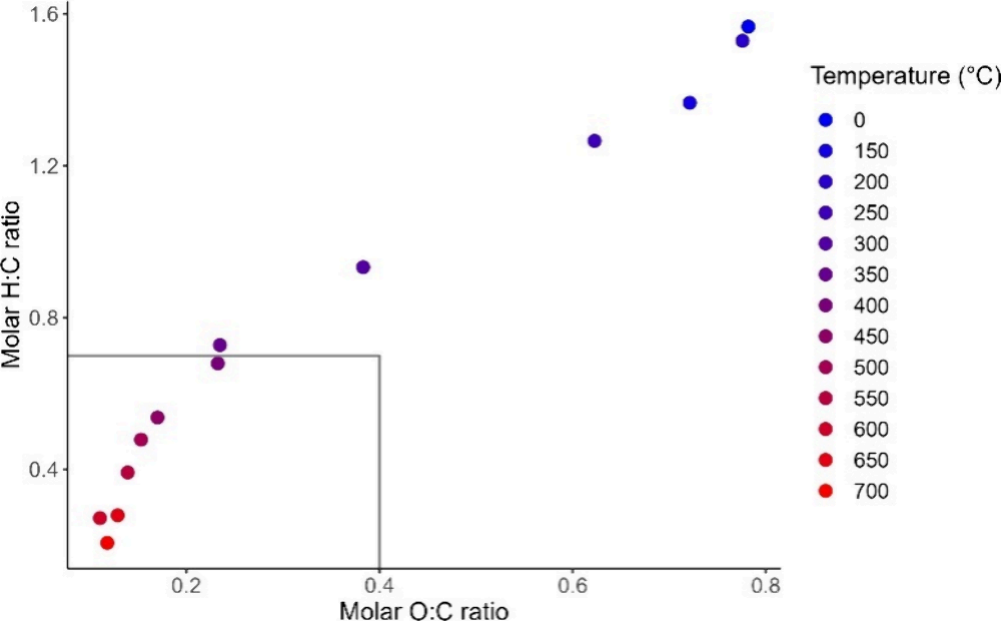
Van Krevelen diagram of barley straw-derived biochar at various pyrolysis temperatures comparing molar H:C ratios with molar O:C ratios. The rectangle encompasses the ratios required for biochar to be certified and later used in carbon offsetting schemes.

Van Krevelen diagrams have become a common way among biochar researchers to illustrate both H:C and O:C ratios while comparing feedstocks or temperatures and quickly determining if biochar composition adheres to accreditation criteria. Figure 3 demonstrates a clear trend of reduction of both H:C and O:C with temperature in an S-shaped curve, with most drastic changes in molar ratios occurring in the middle range temperatures and more incremental changes occurring at the extremes. All biochars produced at ≥400 °C are closely clustered and fall within the IBI^23^ and EBC^24^ guidelines of H:C < 0.7 and O:C < 0.4, indicative of biochar suitable for soil application. A published two-component model^43^ has proposed that biochars with H:C < 0.7 retain 50% of their carbon after 100 years (BC_+100_) and those with H:C < 0.4 retain 70% over the same time period. According to these criteria, the biochars produced in this study at ≥400 °C have achieved BC_+100_ of 50%, and those produced at ≥550 °C attained BC_+100_ of 70%. According to previously published O:C ratio modeling,^41^ the biochar produced at ≥300 °C has a half-life of 100 years (O:C < 0.4), and biochar produced at ≥450 °C has a half-life of 1000 years (O:C < 0.2).

### FTIR Data

**Figure 4:**
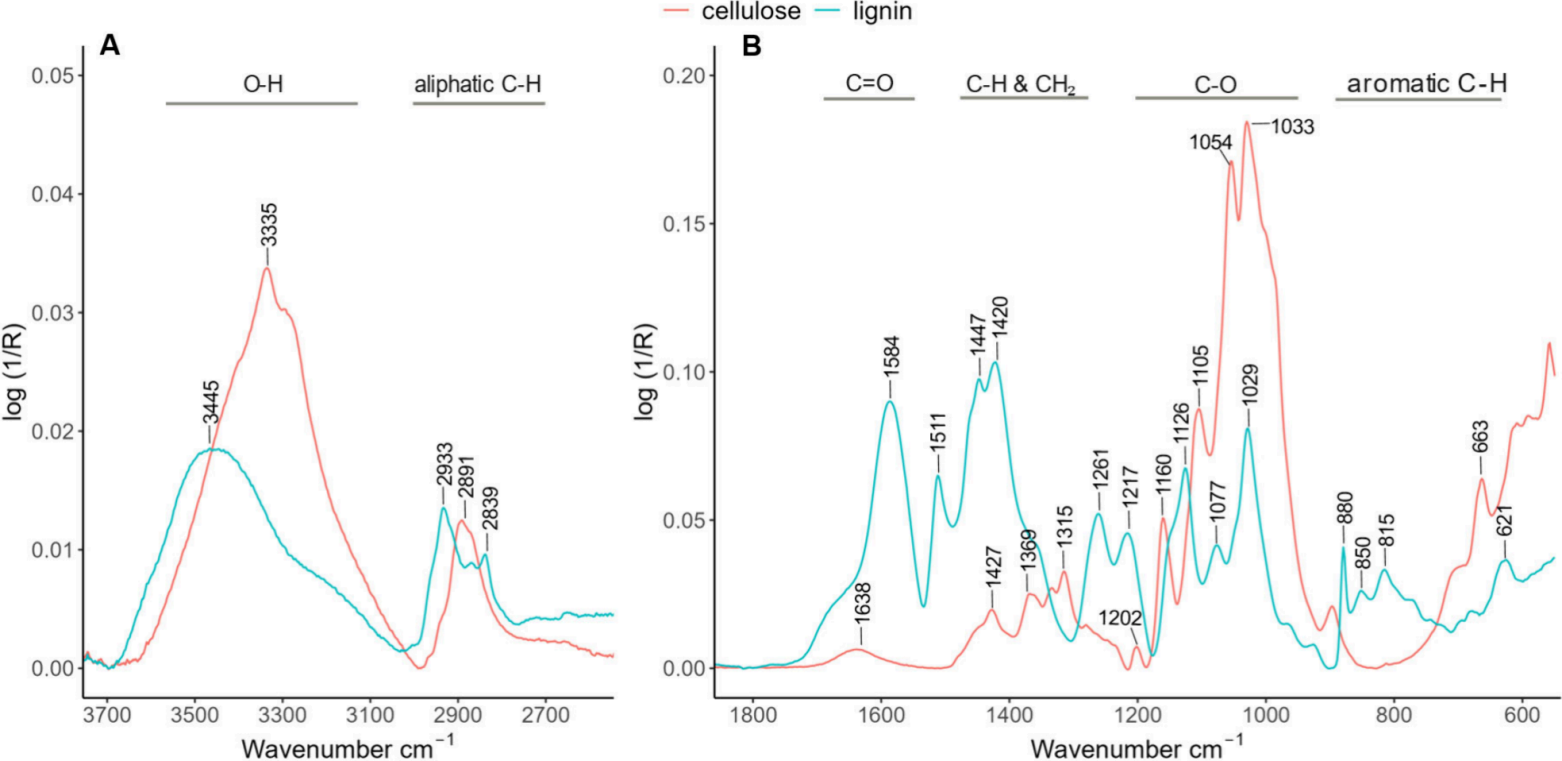
ATR-FTIR of cellulose and kraft lignin, with six replicates averaged for illustrative simplicity. *Y*-axis is on a common scale. (A) The region from 3700 to 2600 cm^–1^ and (B) the fingerprint region from 1800 to 550 cm^–1^. Notable peaks are labeled at their wavenumbers, and chemical assignments are available in Table 1.

**Figure 5:**
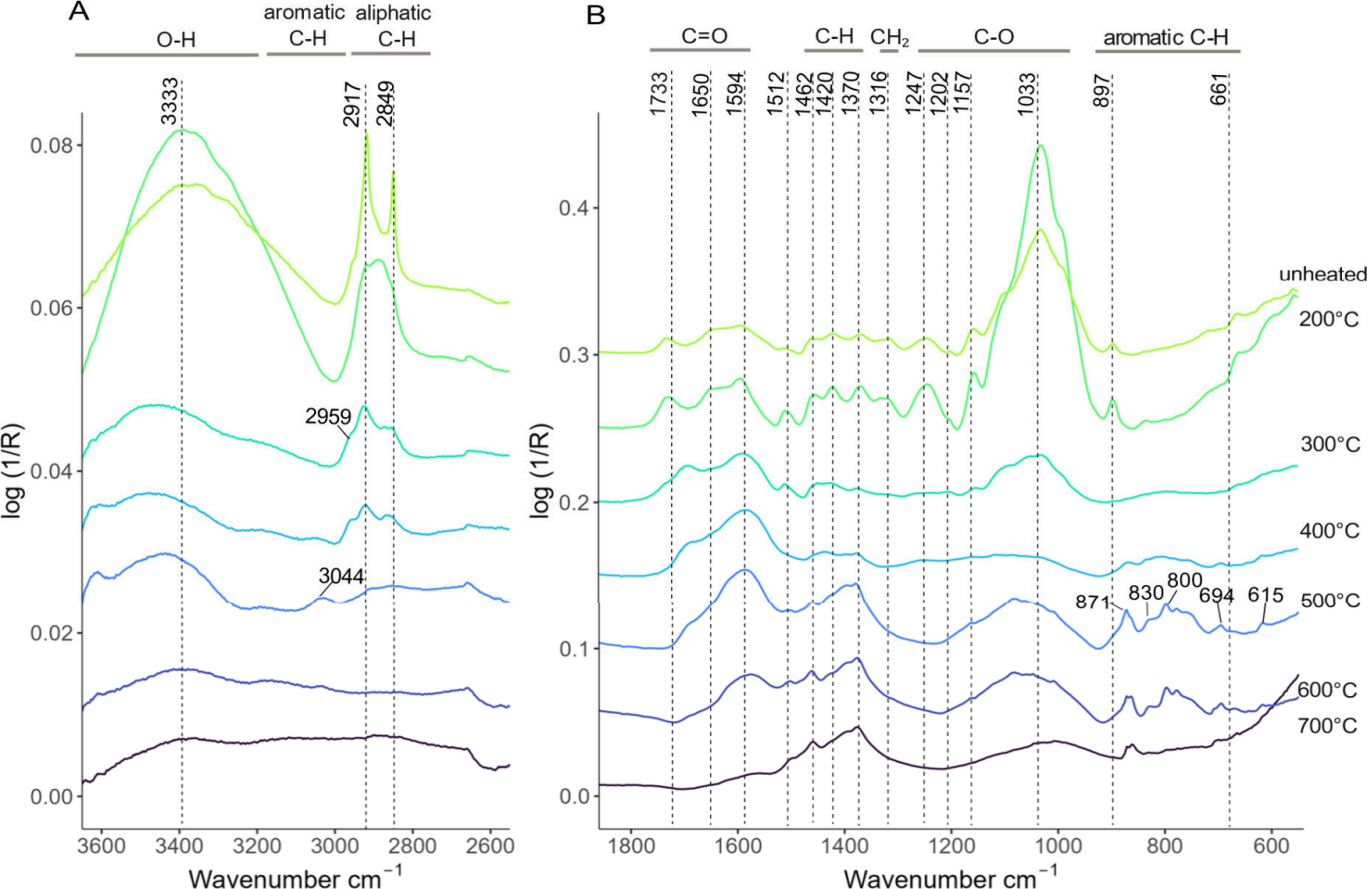
ATR-FTIR of barley straw-derived biochars at various pyrolysis temperature treatments. (A) The region from 3600 to 2600 cm^–1^ and (B) the fingerprint region from 1800 to 550 cm^–1^. The *Y*-axis is offset on a common scale, and each line is a baselined average of six FTIR replicates, for illustrative simplicity, at each temperature treatment. Notable peaks are labeled at their wavenumbers, and chemical assignments are available in Table 1.

The FTIR spectra of lignin and cellulose, the primary biopolymer constituents of barley straw, and spectra of biochars produced in increments of 100 °C can be found in Figure 4 and Figure 5, respectively. The chemical assignments and references for FTIR peaks present in both Figure 4 and Figure 5 are detailed in Table 1.

**Table 1:** Barley Straw ATR-FTIR Spectral Peaks and Chemical Assignments

Peak (cm^–1^)	Chemical assignment	Refs
3333	H-bonded O–H stretch	(44,45)
3044	Aromatic C–H stretch	(47)
2959	Aromatic C–H stretch	(44)
2917	Alkane C–H stretch	(44,46)
2849	Alkane C–H stretch	(44)
1733	C=O stretch	(49)
1650	C=O stretch	(49)
1594	Aromatic skeletal vibration of lignin and C=O stretching	(44,46)
1512	Aromatic skeletal vibrations of lignin	(44,46,48)
1462	C–H bend and CH_2_ wagging	(44,46,48)
1420	Aromatic skeletal vibration of lignin	(44,46,48)
1370	C–H deformation (sym.) and O–H bending vibrations	(49,58)
1316	C–H bend and CH_2_ wagging	(44)
1247	C–O stretch in guaiacyl unit of lignin	(44,48,49)
1202	Bending C–O–H deformation in-plane at C-6 of cellulose	(58,59)
1157	Stretch (assym.) C–O–C at β-glucosidic linkage in cellulose	(44,58,59)
1033	C–O primary alcohol stretch in cellulose and lignin	(48)
897	C–O–C stretch at β-glycosidic linkage of cellulose	(58)
871	Aromatic C–H	(55,56)
830	C–H bend of syringyl unit of lignin	(60)
800	Aromatic C–H wag	(55,56)
694	Cis-isomers of olefins	(61)
661	C–OH out-of-plane bending	(58)

#### Lignin and Cellulose

Figure 4A reveals the presence of water, implied by the broad O–H stretch at 3600–3000 cm^–1^,^44,45^ as well as aliphatic C–H stretch signals^45,46^ in both biopolymers. The lignin spectra also feature aromatic C–H stretching at 3044 cm^–1^ due to aromatic rings present in the subunits of lignin.^47^ The fingerprint region of cellulose in Figure 4B is dominated by a C–O primary alcohol stretch peak at 1033 cm^–1^,^44,48^ with smaller C–H peaks present at 1470–1370 cm^–1^, consistent with the structure of cellulose.^44^ Notable peaks in the fingerprint region of lignin include carbonyl stretching at 1650 cm^–1^,^49^ aromatic skeletal vibrations from 1512 to 1420 cm^–1^,^44,46,48^ C–H and CH_2_ bending and wagging from 1462 to 1316 cm^–1^,^44,46,48^ and C–O stretch peaks from 1247 to 1033 cm^–1^.^44,48,49^

#### Biochar Spectra

Figure 5 clearly illustrates the inverse relationship between the temperature at which biochar is produced and the presence of functional groups, with high-temperature biochars exhibiting limited functionality. Lignin and cellulose are prominent in the spectra of the unpyrolyzed barley straw. In Figure 5A, the level of the O–H stretch peaks decreases most from 200 to 300 °C, indicating dehydration, which corresponds to the mass loss at this temperature observed in Figure 2A and the great reduction of oxygen and hydrogen occurring in this temperature range in Figure 2B. Aromatic C–H stretch signals at 2959 and 3044 cm^–1^ weakly appear at 300 and 500 °C, respectively, but are otherwise not present. Several studies that use FTIR to determine aromaticity in coal rely on this aromatic C–H signal,^50−53^ but these results suggest this region alone is not reliable for quantifying aromaticity in biochar. Aliphatic C–H stretch signals are prominent in unpyrolyzed barley, remain present until 400 °C, and then disappear at higher temperatures.

In Figure 5B, several carbonyl signals exist in the range 1750–1550 cm^–1^ at lower temperatures but then merge into one peak at 1594 cm^–1^ at 500 °C, signifying the loss of oxygen from the biochars.^45^ Peaks of C–H bending and wagging also gradually reduce with temperature, leaving only C–H deformation (sym.) at 1370 cm^–1^ remaining at 700 °C. The CH_2_ wagging signals at 1316 cm^–1^ disappear at 300 °C. There is an increase of C–O stretch peak intensity at 1033 cm^–1^ from unpyrolyzed barley straw to 200 °C, attributed to the breaking of C=O bonds.^54^ This is followed by a dramatic reduction at 300 °C and then a slight increase from 500 to 700 °C caused by phenol and furan structures formed by rearrangements.^45^ Lastly, signals in the region 900–660 cm^–1^ indicative of aromatic C–H^55,56^ are present at 500 and 600 °C but are lost at 700 °C, likely due to the biochar becoming comprised mostly of graphite with diminished functionality.^57^ For FTIR spectra of biochar produced at all pyrolysis temperatures, see Figure S1.

### Modeling

Two distinct models for predictions of H:C and O:C were evaluated with various spectral pre-processing and regression methods, the results of which are given in Table 2. Briefly, the H:C models produced *R*^2^ values in the range 0.79–0.99, MAPE values from 6.36 to 30.54%, and RMSE of 0.05–0.24. The O:C model produced *R*^2^ values in the range 0.79–0.99, MAPE values from 8.36 to 37.46%, and RMSE of 0.03–0.13. The similar *R*^2^ ranges for both molar ratios suggest that regression modeling is equally suitable for predicting either H:C or O:C, and either independent model would be a sufficient indicator of stability. However, the slightly higher MAPE range for the O:C model implies that predictions have larger discrepancies from actual values for this molar ratio. This could be due to more variance in the FTIR peaks associated with C–O and C=O bonding (Figure 5), which have the largest intensities, as compared to C–H and CH_2_ signals.

**Table 2:** Model Testing Results, Sorted by Descending *R*^2^: (A) H:C Prediction Results on Test Data Set and (B) O:C Prediction Results on Test Data Set^a^

Model version	Spectrum	Method	Normalized	Scaled	RMSE	MAPE (%)	*R*^2^
**(A) H:C Prediction Results**							
1	Fingerprint	PLSR	√	√	0.05	7.01	0.99
2	Fingerprint	PLSR	√		0.05	8.17	0.99
3*	Cleaned	PLSR	√		0.07	6.86	0.99
4	Whole	PLSR	√		0.07	7.71	0.99
5	Fingerprint	PCR	√	√	0.06	6.36	0.98
6	Cleaned	PLSR	√	√	0.08	13.68	0.98
7	Whole	PLSR	√	√	0.08	13.06	0.98
8	Fingerprint	PCR	√		0.08	11.86	0.98
9	Cleaned	PCR	√		0.08	10.88	0.97
10	Whole	PCR	√		0.09	10.52	0.97
11	Cleaned	PCR	√	√	0.11	14.08	0.95
12	Whole	PCR	√	√	0.12	15.31	0.94
13	Fingerprint	PLSR			0.17	14.97	0.94
14	Whole	PLSR			0.17	18.24	0.94
15	Cleaned	PLSR		√	0.16	21.39	0.93
16	Cleaned	PLSR			0.18	15.83	0.93
17	Fingerprint	PLSR		√	0.19	15.95	0.92
18	Whole	PLSR		√	0.16	30.54	0.91
19	Whole	PCR			0.19	22.47	0.87
20	Cleaned	PCR			0.19	22.34	0.87
21	Fingerprint	PCR			0.21	21.01	0.84
22	Fingerprint	PCR		√	0.21	19.85	0.83
23	Cleaned	PCR		√	0.22	26.90	0.82
24	Whole	PCR		√	0.24	29.74	0.79

**(B) O:C Prediction Results**							
1*	Cleaned	PLSR	√		0.03	9.02	0.99
2	Whole	PLSR	√		0.03	8.77	0.99
3	Fingerprint	PLSR	√		0.03	11.16	0.99
4	Fingerprint	PLSR	√	√	0.02	8.36	0.99
5	Fingerprint	PCR	√	√	0.03	10.43	0.99
6	Cleaned	PLSR	√	√	0.04	14.53	0.98
7	Whole	PLSR	√	√	0.04	16.59	0.98
8	Fingerprint	PCR	√		0.04	16.12	0.98
9	Cleaned	PCR	√		0.04	14.62	0.98
10	Whole	PCR	√		0.04	15.15	0.97
11	Cleaned	PCR	√	√	0.05	18.96	0.96
12	Whole	PCR	√	√	0.06	22.05	0.95
13	Cleaned	PLSR		√	0.08	23.82	0.94
14	Whole	PLSR			0.09	18.60	0.93
15	Fingerprint	PLSR			0.09	18.71	0.93
16	Fingerprint	PLSR		√	0.10	17.94	0.93
17	Cleaned	PLSR			0.10	15.72	0.93
18	Whole	PLSR		√	0.08	35.22	0.91
19	Whole	PCR			0.10	26.72	0.88
20	Cleaned	PCR			0.10	26.41	0.87
21	Fingerprint	PCR			0.11	26.20	0.84
22	Fingerprint	PCR		√	0.11	23.95	0.84
23	Cleaned	PCR		√	0.12	32.93	0.83
24	Whole	PCR		√	0.13	37.46	0.79

a Model versions used in final predictions are denoted with *. Spectrum descriptions are available in the Materials and Methods section. PLSR: partial least squares regression, PCR: principal component regression, RMSE: root mean squared error, MAPE: mean absolute percent error.

PLSR generally outperformed PCR, likely due to its supervised nature. The top 50% of model versions for both H:C and O:C predictions utilized normalized spectra, strongly suggesting that normalization of FTIR data produces more accurate predictions. Scaling the data had mixed results, potentially due to a loss of information that is useful in the prediction. Models that combine scaling and normalization achieved better *R*^2^ values than those using scaling alone. Sub-setting the spectra did not appear to have a substantial effect on final prediction compared to other pre-processing steps and therefore may not be necessary. The final deployed models used to predict molar ratios (denoted with an asterisk in Table 2) retained 8 components and utilized the cleaned version of the spectrum, with normalized but unscaled data and a PLSR algorithm.

#### Comparison of Measured and Predicted Molar Ratios

Correlation plots of measured and predicted molar ratios individually can be found in Figure S2. The best H:C model produced an *R*^2^ of 0.99, MAPE of 6.86%, and RMSE of 0.07. The best O:C model achieved the same *R*^2^ (0.99), but with slightly more error than the H:C model: MAPE 9.02% and RMSE 0.03. In both cases the model was slightly better at predicting lower values, and error is mostly attributed to higher values which are from unpyrolyzed material or pyrolysis temperature ≤250 °C. This could be due in part to sample heterogeneity causing more variance in the FTIR results.

**Figure 6:**
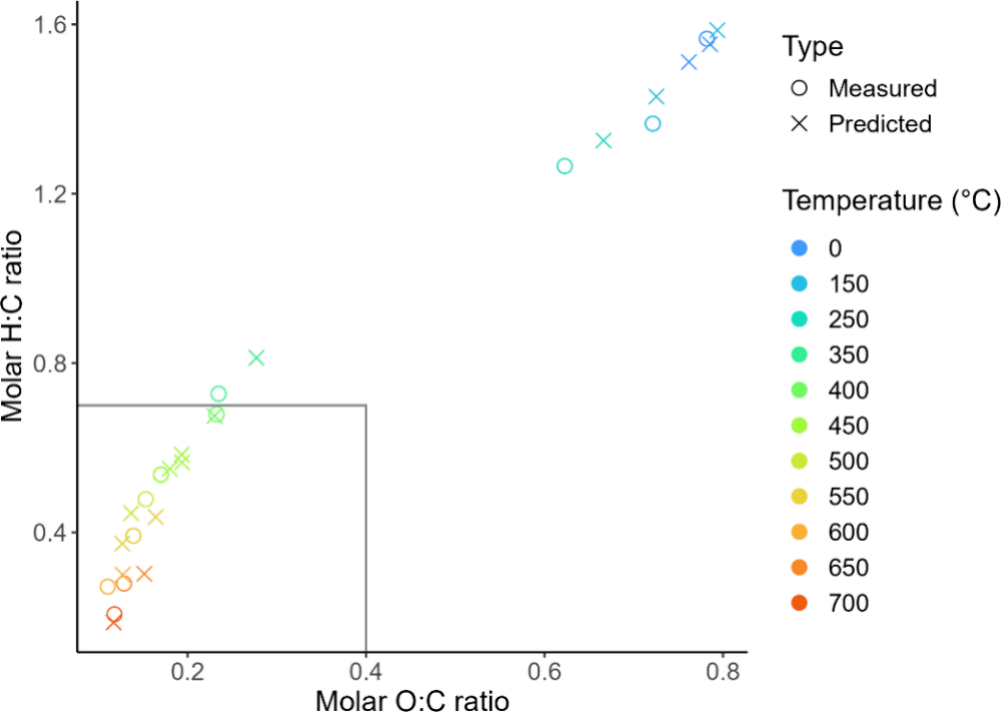
Comparison of measured H:C and O:C values with those predicted by the PLSR model. Only samples randomized to the “test” set are included, meaning modeling results are not inclusive of all temperatures. The elemental analysis data have a relative standard deviation for the instrument of 2%, which is smaller than the size of the symbols.

The close correlation between the measured molar ratios and the model’s predictions is illustrated in the van Krevelen plot in Figure 6. In the region where H:C < 0.7 and O:C < 0.4, the predictions nearly directly overlap with the measured molar ratios, meaning the model can very reliably predict stability indices at high-temperature treatments. Because of the randomization of samples, biochar produced at 300 °C was not included in the test set, and thus further modeling studies should explore this temperature. The success of the model’s predictions signifies that it is a promising method for attaining stability data without the need of expensive elemental analysis.

Though there are no studies to date combining FTIR spectral data with PLSR to determine molar ratios of biochar, studies using NIR spectroscopy found similar success in their predictions. Both Munawar et al.^34^ and Xie et al.^35^ performed PLSR modeling using NIR to predict other biochar properties, such as fixed carbon, volatile matter, and ash, and observed *R*^2^ values from 0.80 to 0.86 and from 0.93 to 0.95, respectively, but both studies failed to link this work to molar ratios. Kusumo et al.^33^ built a PLSR model that achieved an H:C prediction with *R*^2^ = 0.94 and O:C with *R*^2^ = 0.89. Though our FTIR-based model has produced slightly better results than their NIR model, the discrepancies could be attributed to experimental design, as that study analyzed six feedstock groups at five temperature treatments, whereas this study analyzed only one feedstock over 13 pyrolysis temperatures.

#### PLSR Model Loadings

**Figure 7:**
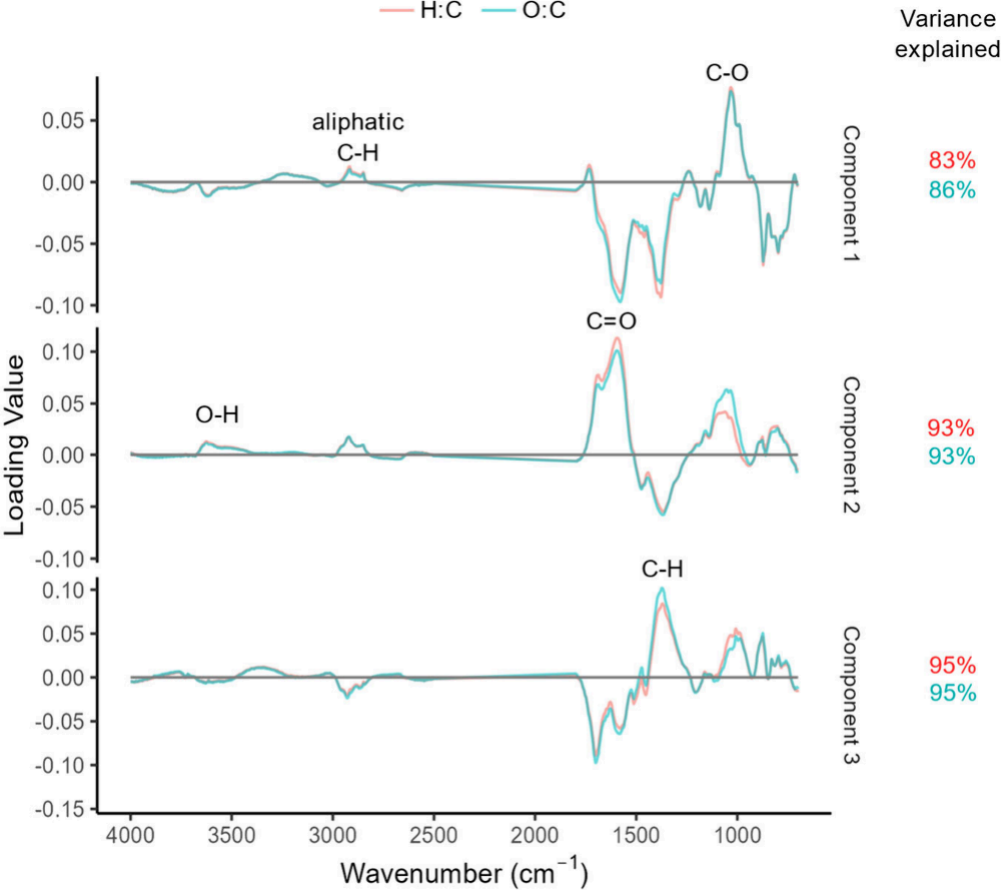
Loadings of the first three components of both the H:C and O:C models, with respective variance explained. Chemical assignments of the most influential regions on the predictions are labeled (see Table 1 for more detail).

In order to determine which regions of the spectra were most influential in prediction, the model loadings were explored in Figure 7. Positive peaks are correlated with H:C and O:C ratios, with higher values suggestive of raw feedstocks or partially pyrolyzed biomass, whereas negative loadings signify an inverse correlation with both ratios and indicate more stable biochar. The loadings for the two individual models are nearly identical, with small discrepancies in the intensities or shapes of the peaks. This suggests that both models rely on essentially the same set of chemical signals, and therefore, either model would be sufficient individually to predict stability. It should be noted that, due to pre-processing of FTIR data, the region 2500–1800 cm^–1^ was removed and therefore does not contribute to model loadings.

The first component explains 83% (H:C) and 86% (O:C) of the variance of the models’ predictions. Peaks in the O–H stretch region of 3600–3000 cm^–1^ and the aliphatic C–H stretch in 3000–2800 cm^–1^ have limited influence on prediction. This is likely because these signals are completely removed at 400 °C (Figure 5A). The regions associated with C=O stretch, approximately 1750–1550 cm^–1^, and C–H bending and wagging, 1470–1370 cm^–1^, were inversely correlated with the model’s prediction of the molar ratios. This suggests that the absence of C=O- and C–H-containing functional groups is indicative of lower H:C and O:C values and, therefore, more stable biochar within this first component. Finally, the C–O stretch region at 1030 cm^–1^ is largely impactful and positively correlated with H:C and O:C. This is expected, as the lowest pyrolysis temperatures corresponded with the highest H:C and O:C values (Figure 3) and the largest peaks in this region (Figure 5B).

The second component was then created on the residual data remaining after the first component and therefore independently explains only about 10% of the variance. Interestingly, in the second component, C=O peaks become positively correlated with H:C and O:C, meaning that there is a nonlinear relationship between this region and the predicted molar ratios. The second component also reveals a stronger influence in the C–O stretch region 1200–1000 cm^–1^ on the O:C model as compared to the H:C model. In the third component, the C=O region is again negatively correlated with H:C and O:C as it was in the first. This complicated relationship suggests that it is not advisable to rely on this peak alone as an indicator for H:C or O:C. The same pattern is observed on the C–H bending peak, where the loading is negative in components 1 and 2, but positive in component 3. The only region consistently associated with higher H:C and O:C values is the C–O signal. Therefore, for a simplistic indicator of stability, it is suggested to look for diminishment of the C–O stretch peak in the 1200–1000 cm^–1^ region of biochar spectra.

## Conclusions

Due to time and cost constraints, a limitation of this study is that only one sample of biochar was produced at each temperature, and future studies would benefit from replicates in temperature treatments. This would allow for more training data to be available for the model. Additionally, though the initial modeling results appear promising using only one homogeneous feedstock, future evaluation of the model’s success with other feedstocks will be essential to understand the applicability of this method on a wider range of diverse starting materials. For future studies using FTIR data combined with predictive modeling, it is advised to use normalized spectra with a PLSR algorithm for best results. The use of statistical models using FTIR data could also be broadened to predict other stability metrics such as volatile matter, fixed carbon, and ash content. Overall, the results suggest that the use of FTIR data is a promising new method for quantitatively determining the H:C and O:C ratios of biochar. As FTIR is a rapid, non-destructive, and relatively accessible analytical tool already in use in biochar research, results of this study signify that stability proxies such as H:C and O:C ratios can become more easily available to the biochar community.

## Supporting Information Available

Figure S1, comprehensive figure of FTIR spectra for all samples; Figure S2, correlation plots of measured vs predicted elemental ratios (PDF)
All FTIR spectral data obtained in this work (XLSX)
