# Supplementary material for: Predicting Stability of Barley Straw-Derived Biochars Using Fourier Transform Infrared Spectroscopy
Source: ACS Sustain Resour Manag. 2024 Aug 16;1(9):1975–83. doi: 10.1021/acssusresmgt.4c00148 (PMC11449111; doi:10.1021/acssusresmgt.4c00148)

Supporting Information

Predicting Stability of Barley Straw-Derived Biochars Using Infrared Spectroscopy (FTIR)

Authors: Monica A. McCall * †,‡, Jonathan S. Watson †, and Mark A. Sephton †

†Earth Science and Engineering, Imperial College London, Exhibition Rd, South Kensington, London, SW7 2BX, United Kingdom

‡Grantham Institute for Climate Change and the Environment, Imperial College London, South Kensington, London, SW7 2AZ, United Kingdom

*Corresponding author: monica.mccall@imperial.ac.uk

Table of Contents

Article title 1

Authors 1

Data 2

Figure S1 2

Figure S2 3

Data

All FTIR spectral data used in this study can be found in the file FTIR_data.xlsx.

Figure S1

ATR-FTIR of barley straw biochars at all peak temperature treatments; A) the region from 3600-2600 cm-1, B) the fingerprint region from 1800-600 cm^{-1} Y-axis is offset on a common scale, each line is a baselined average of six FTIR runs at each temperature treatment.

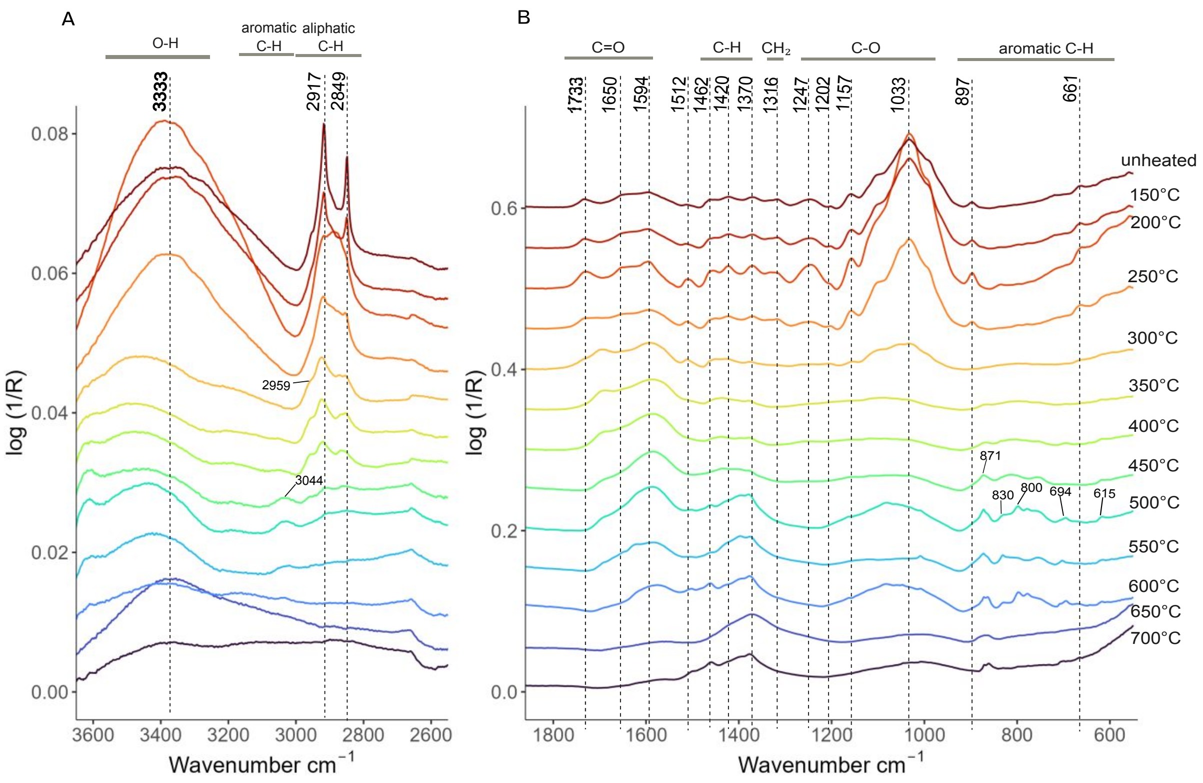

Figure S2

Measured molar ratios compared to those predicted by the PLSR model, A) H:C ratio, B) O:C ratio. Prediction only included samples randomized to the "test" set and are not inclusive of all temperatures.

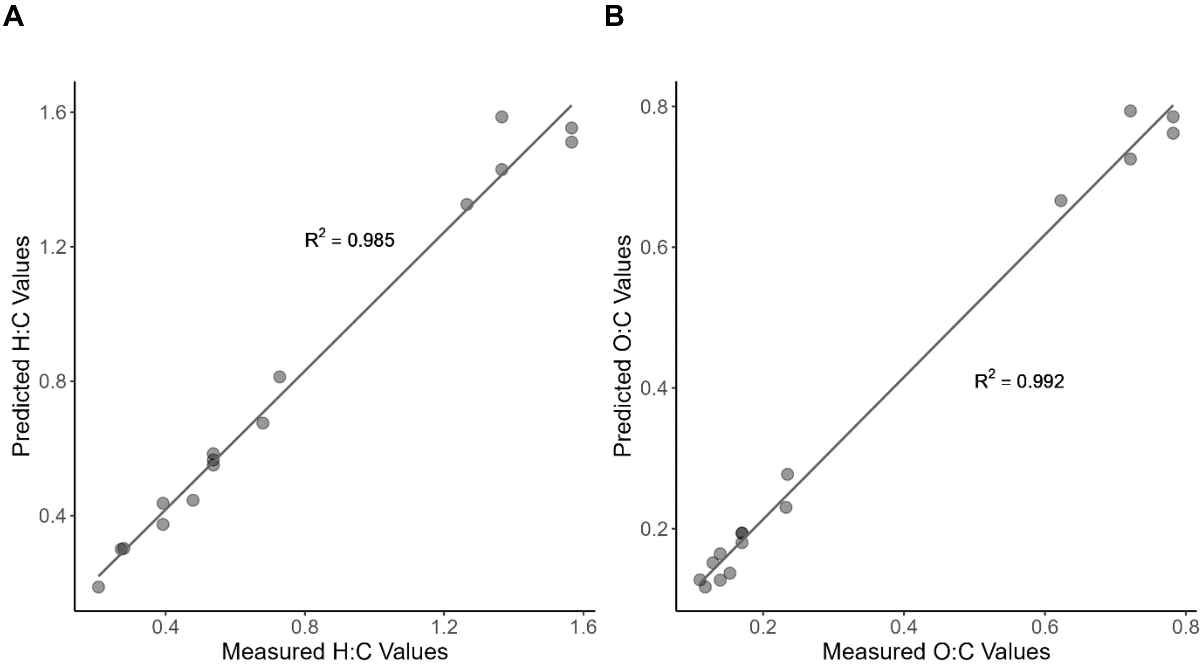

Supplement: Supplementary file 1 — rm4c00148_si_002.pdf [file rm4c00148_si_002.pdf]
